# Unravelling the complexity of ventilator-associated pneumonia: a systematic methodological literature review of diagnostic criteria and definitions used in clinical research

**DOI:** 10.1186/s13054-024-04991-3

**Published:** 2024-07-02

**Authors:** Markus Fally, Faiuna Haseeb, Ahmed Kouta, Jan Hansel, Rebecca C. Robey, Thomas Williams, Tobias Welte, Timothy Felton, Alexander G. Mathioudakis

**Affiliations:** 1 Department of Respiratory Medicine and Infectious Diseases, Copenhagen University Hospital – Bispebjerg and Frederiksberg, Copenhagen, Denmark; 2 North West Lung Centre, Wythenshawe Hospital, Manchester University NHS Foundation Trust, Manchester Academic Health Science Centre, Manchester, UK; 3 Division of Immunology, Immunity to Infection and Respiratory Medicine, School of Biological Sciences, The University of Manchester, Manchester, UK; 4 North West School of Intensive Care Medicine, Health Education England North West, Manchester, UK; 5 Acute Intensive Care Unit, Wythenshawe Hospital, Manchester University NHS Foundation Trust, Manchester Academic Health Science Centre, Manchester, UK; 6 Department of Respiratory Medicine and German Centre of Lung Research (DZL), Hannover Medical School, Hannover, Germany

**Keywords:** Diagnostic criteria, Inclusion criteria, Clinical trial, Ventilator-associated pneumonia, Systematic review

## Abstract

**Background:**

Ventilator-associated pneumonia (VAP) is a prevalent and grave hospital-acquired infection that affects mechanically ventilated patients. Diverse diagnostic criteria can significantly affect VAP research by complicating the identification and management of the condition, which may also impact clinical management.

**Objectives:**

We conducted this review to assess the diagnostic criteria and the definitions of the term “ventilator-associated” used in randomised controlled trials (RCTs) of VAP management.

**Search methods:**

Based on the protocol (PROSPERO 2019 CRD42019147411), we conducted a systematic search on MEDLINE/PubMed and Cochrane CENTRAL for RCTs, published or registered between 2010 and 2024.

**Selection criteria:**

We included completed and ongoing RCTs that assessed pharmacological or non-pharmacological interventions in adults with VAP.

**Data collection and synthesis:**

Data were collected using a tested extraction sheet, as endorsed by the Cochrane Collaboration. After cross-checking, data were summarised in a narrative and tabular form.

**Results:**

In total, 7,173 records were identified through the literature search. Following the exclusion of records that did not meet the eligibility criteria, 119 studies were included. Diagnostic criteria were provided in 51.2% of studies, and the term “ventilator-associated” was defined in 52.1% of studies. The most frequently included diagnostic criteria were pulmonary infiltrates (96.7%), fever (86.9%), hypothermia (49.1%), sputum (70.5%), and hypoxia (32.8%). The different criteria were used in 38 combinations across studies. The term “ventilator-associated” was defined in nine different ways.

**Conclusions:**

When provided, diagnostic criteria and definitions of VAP in RCTs display notable variability. Continuous efforts to harmonise VAP diagnostic criteria in future clinical trials are crucial to improve quality of care, enable accurate epidemiological assessments, and guide effective antimicrobial stewardship.

**Supplementary Information:**

The online version contains supplementary material available at 10.1186/s13054-024-04991-3.

## Background

Ventilator-associated pneumonia (VAP) stands as the most prevalent and serious hospital-acquired infection observed in intensive care units [1]. VAP prolongs hospital stays, durations of mechanical ventilation, and is associated with considerable mortality and an increase in healthcare costs [2, 3].

Diagnosing VAP can be challenging for clinicians as it shares clinical signs and symptoms with other forms of pneumonia as well as non-infectious conditions [4]. The most recent international clinical guidelines define VAP as the presence of respiratory infection signs combined with new radiographic infiltrates in a patient who has been ventilated for at least 48 h [5, 6]. While the guidelines developed by ERS/ESICM/ESCMID/ALAT do not provide a detailed definition of signs of respiratory infection [5], the ATS/IDSA guidelines mention that clinical signs may include the new onset of fever, purulent sputum, leucocytosis, and decline in oxygenation [6]. However, the ATS/IDSA guideline panel also acknowledges that there is no gold standard for the diagnosis of VAP [6]. This lack of a standardised definition is further highlighted by the varying, surveillance-based definitions of VAP provided by the Centre for Disease Control (CDC) and the European Centre for Disease Control (ECDC) [7, 8]. These definitions, focusing on a combination of clinical, radiological, and microbiological signs to identify cases of VAP, were established to standardise reporting and facilitate the monitoring of infections in healthcare settings. However, the criteria given by the CDC and ECDC may not always align with the diagnostic criteria used by clinicians to confirm or rule out the condition [9–11].

Variations in the eligibility criteria applied to VAP can have a significant impact on systematic reviews and meta-analyses that assess different interventions, primarily due to the potential lack of comparability among the studied populations [12]. Furthermore, the incidence of VAP may be underestimated when excessively strict diagnostic criteria are employed [13, 14].

A recent systematic review conducted by Weiss et al. focused on inclusion and judgment criteria used in randomised controlled trials (RCTs) on nosocomial pneumonia and found considerable heterogeneity [15]. However, the authors only considered RCTs evaluating antimicrobial treatment as interventions, did not distinguish between hospital-acquired pneumonia (HAP) and VAP, and did not evaluate definitions of the term "ventilator-associated".

The objective of this systematic review was to provide a concise overview of the diagnostic criteria for VAP recently used in RCTs, as well as the definitions attributed to the term "ventilator-associated". Its findings will provide valuable insights to a forthcoming task force, which aims to establish a uniform definition and diagnostic criteria for VAP in clinical trials. The task force will be made up of representatives from prominent international societies with an interest in VAP, as well as patient partners with lived experience. The harmonisation of the diagnostic criteria for VAP in upcoming clinical research are vital for enhancing patient care, enabling accurate epidemiological studies, and guiding successful antimicrobial stewardship programs.

## Methods

### Protocol and registration

The protocol for this systematic review was registered in advance with the International Prospective Register of Systematic Reviews (PROSPERO 2019 CRD42019147411), encompassing a broad review focusing on pneumonia outcomes and diagnostic criteria in RCTs. Recognising the limitations of discussing all findings in one manuscript, we opted to produce several focused and comprehensive manuscripts, all employing the same fundamental methodology, as registered with PROSPERO. While a previous publication focused on outcomes reported in RCTs on pneumonia management [16], the current submission specifically addresses diagnostic criteria for VAP.

### Eligibility criteria

We included RCTs that were registered, planned, and/or completed that: (1) enrolled adults with VAP; and (2) assessed the safety, efficacy and/or effectiveness of pharmacological or non-pharmacological interventions for treating VAP.

We have excluded systematic reviews, meta-analyses, narrative reviews, post hoc analyses from RCTs, observational studies, case reports, editorials, conference proceedings, and studies that do not exclusively focus on pneumonia (such as trials including patients with pneumonia alongside other diseases). Additionally, studies on pneumonia subtypes other than VAP, such as pneumonia without specifying a subtype, community-acquired pneumonia (CAP), healthcare-associated pneumonia (HCAP), and HAP, have also been excluded. To maintain focus and relevance, studies on Coronavirus Disease 2019 (COVID-19) were excluded from this systematic review, as the viral aetiology and distinct clinical management protocols differ significantly from the nature and treatment strategies of VAP. RCT protocols were only included if the results have not been previously published in another article included in this systematic review. Due to resource constraints and the lack of multilingual expertise within the review team, this systematic review was restricted to English-language RCTs.

### Information sources and search

On 20 May 2024, we searched MEDLINE/PubMed, and the Cochrane Register of Controlled Trials (CENTRAL) for RCTs published between 1 January 2010 and 19 May 2024. We used electronic algorithms introducing a combination of controlled vocabulary and search terms as reported in the Appendix.

### Study selection

Two reviewers (FH, MF) independently screened titles and abstracts to identify eligible studies using Rayyan [17]. In case of disagreement, a third reviewer was consulted (AGM). After immediate exclusion of duplicates using EndNote X9, four reviewers (AGM, FH, JH, MF) independently checked for eligibility at full-text level. The results of the selection process are reported according to the Preferred Reporting Items for Systematic Reviews and Meta-Analyses (PRISMA) [18].

### Data collection process

We developed an extraction sheet as endorsed by the Cochrane Collaboration [19]. The extraction sheet was independently tested by three reviewers (AGM, FH, MF) on five randomly selected studies and adapted to ensure good inter-reviewer agreement. The extraction sheet contained the following elements: (1) study ID, name, reference and NCT number; (2) type of pneumonia: CAP, HCAP, HAP and/or VAP; (3) diagnostic criteria for pneumonia; (4) definition of setting; (5) study origin, design, populations, interventions, and outcomes.

Four reviewers (AGM, FH, JH, MF) extracted data from the eligible studies. Data were extracted sequentially from either a manuscript containing published results, a published protocol, or, upon obtaining a trial registration number from CENTRAL, from one of the designated trial registries, such as ClinicalTrials.gov, the Clinical Trials Registry India (CTRI), the Chinese Clinical Trial Registry (ChiCTR), the European Clinical Trials Database (EudraCT), the Iranian Registry of Clinical Trials (IRCT), the Japan Primary Registries Network (JPRN), and the Japanese University Hospital Medical Information Network Clinical Trials Registry (UMIN-CTR). Cross-checking of all extracted data was performed by a second reviewer (AGM, AK, MF, RR, TW). Disagreements regarding data collection were resolved by discussion between all reviewers.

### Synthesis of results

The findings were consolidated through a combination of narrative and tabular formats. The presentation encompassed the quantitative representation of each diagnostic criterion in terms of numerical values and proportions. Additionally, we provide an analysis of the various combinations of diagnostic criteria employed in RCTs in a sunburst diagram and a tabular format, along with an examination of the definitions attributed to the term "ventilator-associated".

### Risk of bias

The main goal of this systematic review was to explore the diagnostic criteria used in clinical trials for diagnosing VAP. It covered trials with published protocols and/or results, as well as those only registered in a trial database. The varying levels and gaps in the information provided by the various sources made it difficult to conduct a reliable and meaningful risk of bias assessment for all included studies. However, for RCTs with published data, risk of bias was evaluated by four reviewers (AGM, JH, MF, RR) using the Risk of Bias in Randomized Trials 2 tool (RoB-2 tool), as endorsed by the Cochrane Collaboration [20].

## Results

### Study selection and characteristics

**Fig. 1:**
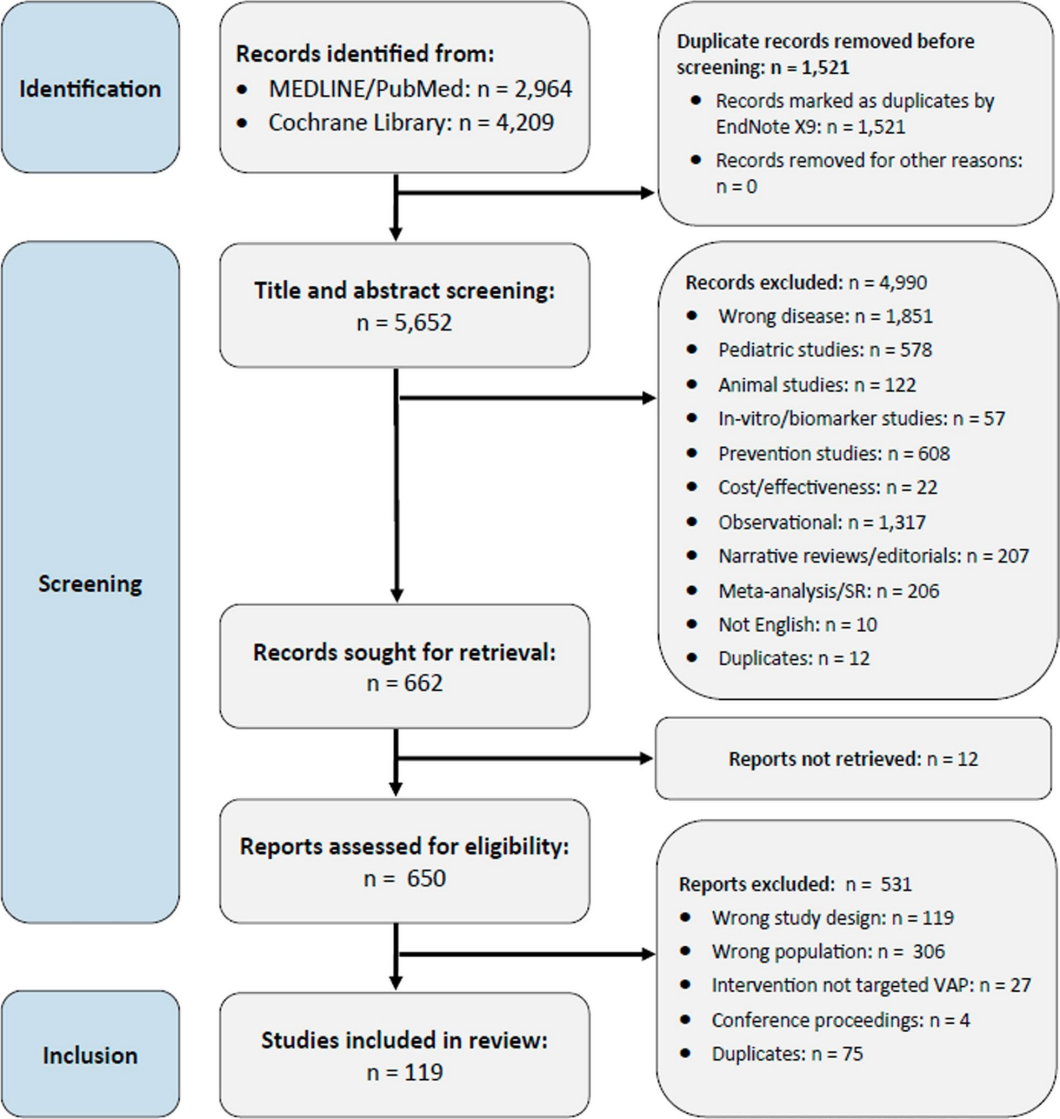
PRISMA flowchart showing study selection

A total of 7173 records were identified through the databases MEDLINE and CENTRAL, as illustrated in Fig. 1. Following the removal of duplicate entries, a screening process involving the evaluation of titles and abstracts was conducted on 5652 records. Among these, 650 records were deemed potentially eligible for inclusion. Ultimately, our review included 119 studies that specifically focused on VAP (Table S1 in the Appendix, the full dataset is available online [21]).

The total number of patients in the 119 identified studies was 21,289. Among these studies, 83 focused exclusively on VAP, while the remaining studies encompassed various subtypes of pneumonia in addition to VAP (see Table 1). The majority of these studies were registered, and their protocols were accessible either through publication in a journal article or on a clinical trial platform. Results were accessible in 56.3% of cases, while both results and the protocol were accessible in 36.9% of cases. In 40.3% of the included studies, data could only be obtained from a trial registry platform, with ClinicalTrials.gov being the primary platform in 36 out of 48 cases, and ChiCTR (n = 2), CTRI (n = 3), EudraCT (n = 3), IRCT (n = 2), JPRN (n = 1) and UMIN-CTR (n = 1) in the remaining cases.

**Table 1:** Characteristics of included reports

VAP	n	%
Studies		
Total	119	
VAP only	83	69.7
HAP, VAP	28	23.5
HAP, HCAP, VAP	4	3.3
CAP, HAP, VAP	3	2.5
CAP, HCAP, HAP, VAP	1	0.8
Registered	98	82.4
Protocol accessible	96	80.7
Results accessible	67	56.3
Protocol and results accessible	44	36.9
Data retrieved from trial registry platform	48	40.3
Patients, in total	21,289	
Patients, median per study	100	
No diagnostic criteria for VAP provided	38	31.9
Referred diagnostic criteria for VAP	20	16.8
Diagnostic criteria for VAP provided	61	51.2
“Ventilator-associated” defined	62	52.1
“Ventilator-associated” not defined	57	47.8

*CAP* community-acquired pneumonia; *HCAP* healthcare-associated pneumonia; *HAP* hospital-acquired pneumonia; *VAP* ventilator-associated pneumonia

Diagnostic criteria were provided in 51.2% and the term “ventilator-associated” was defined in 52.1% of the studies, respectively. Of the 20 studies (16.8%) that referred to previously published diagnostic criteria, 13 cited the Clinical Pulmonary Infection Score (CPIS) [22], while the remaining referred to national and international guidelines.

### Risk of bias

We evaluated the risk of bias in 67 studies with published results using the RoB-2 tool. The overall assessment showed that 25% of the studies were at high risk of bias, 30% were at low risk of bias, and the remaining 45% had some concerns about potential bias. These results indicate variability in the methodological quality of the studies included in the review. The overall risk of bias and the detailed results of our assessments for the 67 studies are displayed in the Appendix (Figures SF1-SF2).

### Diagnostic criteria for VAP

#### Pulmonary infiltrates

Of the 61 studies on VAP that provided diagnostic criteria, 59 (96.7%) included the radiological evidence of a new or progressive pulmonary infiltrate.

#### Clinical signs and symptoms

The most frequently included clinical signs and symptoms were fever (86.9%), hypothermia (49.1%), sputum (70.5%), and hypoxia (32.8%). Different cut-off values were employed to define fever and hypothermia, as indicated in Table 2. The majority of studies, accounting for 45.2%, utilised a cut-off of > 38 degrees Celsius (°C) to define fever, while 13.2% of studies used a cut-off of ≥ 38°C. In the case of hypothermia, the most commonly employed cut-off value was < 35°C, which was utilised in 43.3% of studies that included hypothermia as a criterion. Only a minority of studies provided information on the site of temperature measurement. Oral measurement was the most frequently employed method, followed by axillary and core temperature measurements (further details are displayed in Table S2 in the Appendix).

**Table 2:** Clinical signs and symptoms included in the diagnostic criteria for VAP in RCTs

Clinical signs and symptoms	n	%
*Fever (measured in degrees Celsius)*		
Total	53	86.9
T > 38 °C	24	45.2
T ≥ 38 °C	7	13.2
T > 38.3 °C	4	7.5
T > 38.5 °C	2	3.8
T ≥ 37.5 °C	1	1.9
T > 37.8 °C	1	1.9
T ≥ 38.3 °C	1	1.9
T > 38.4 °C	1	1.9
T > 39 °C	1	1.9
T ≥ 37.5 °C oral, ≥ 37 °C axillary, or ≥ 38 °C rectal	1	1.9
T ≥ 38 °C oral, ≥ 38.3 core/rectal, or ≥ 37.5 °C axillary/forehead	1	1.9
Not further defined	9	17.0
*Hypothermia (measured in degrees Celsius)*		
Total	30	49.2
T < 35 °C	13	43.3
T ≤ 35 °C	7	23.3
T < 35.5 °C	6	20.0
T < 36 °C	2	6.7
T < 36.5 °C	1	3.3
Not further defined	1	3.3
Sputum/expectoration/secretions/aspirate	43	70.5
Hypoxia	20	32.8
*Tachypnoea*		
Total	9	14.8
RR > 30/minute	3	33.3
Not further defined	6	66.6
Dyspnoea	9	14.8
Auscultation/percussion abnormalities	7	11.5
Cough	5	8.2
Clinical signs and symptoms (not further defined)	5	8.2
Bacteria identified	5	8.2
Deteriorating mental or functional status	3	4.9
Respiratory failure	2	3.3
Tachycardia (HR > 120/minute)	2	3.3
Respiratory symptoms (not further defined)	1	1.6
Chest pain/discomfort	1	1.6

*T* temperature; *HR* heart rate; *RR* respiratory rate

#### Biochemistry criteria

Fifty-four studies (88.5%) incorporated white blood count abnormalities as part of their diagnostic criteria for VAP. Conversely, only one study included an elevation of procalcitonin (PCT) as a diagnostic factor, and none of the identified studies included C-reactive protein (CRP). The specific thresholds for leucocytosis and leucopoenia varied across studies, with leucocyte counts ranging from greater than 10,000/mm3 to greater than 12,000/mm3 for leucocytosis, and less than 3,500/mm3 to less than 4,500/mm3 for leucopoenia (Table 3).

**Table 3:** Biochemistry results included in the diagnostic criteria for VAP in RCTs

Biochemistry	n	%
*WBC abnormalities*		
Total	54	88.5
WBC > 10,000/mm^3^ or < 4000/mm^3^ or > 15% bands	12	22.2
WBC > 12,000/mm^3^ or < 4000/mm^3^	12	22.2
WBC > 10,000/mm^3^ or < 4000/mm^3^	8	14.8
WBC > 10,000/mm^3^	4	7.4
WBC > 10,000/mm^3^ or < 4500/mm^3^	3	5.6
WBC > 10,000/mm^3^ or < 4500/mm^3^ or > 15% bands	1	1.9
WBC > 10,000/mm^3^ or < 4000/mm^3^ or > 10% bands	1	1.9
WBC > 12,000/mm^3^	1	1.9
WBC > 11,103/mm^3^	1	1.9
WBC > 11,000/mm^3^ or < 3500/mm^3^	1	1.9
WBC > 11,000/mm^3^ or < 4000/mm^3^	1	1.9
Leucocytosis or leucopoenia (not further defined)	4	7.4
Leucocytosis (not further defined)	5	9.3
*PCT elevation*	1	1.6

*WBC* white blood count; *PCT* procalcitonin

#### Combinations of diagnostic criteria

All definitions of pneumonia were composite in nature and required the fulfilment of a minimum number of predetermined criteria for the diagnosis to be established. In 90.2% of the studies the presence of a new pulmonary infiltrate was a mandatory criterion. Two studies did not include an infiltrate as criterion, whereas the remaining studies (n = 4) included the presence of an infiltrate in their criteria, it was, however, not required for a diagnosis.

**Fig. 2:**
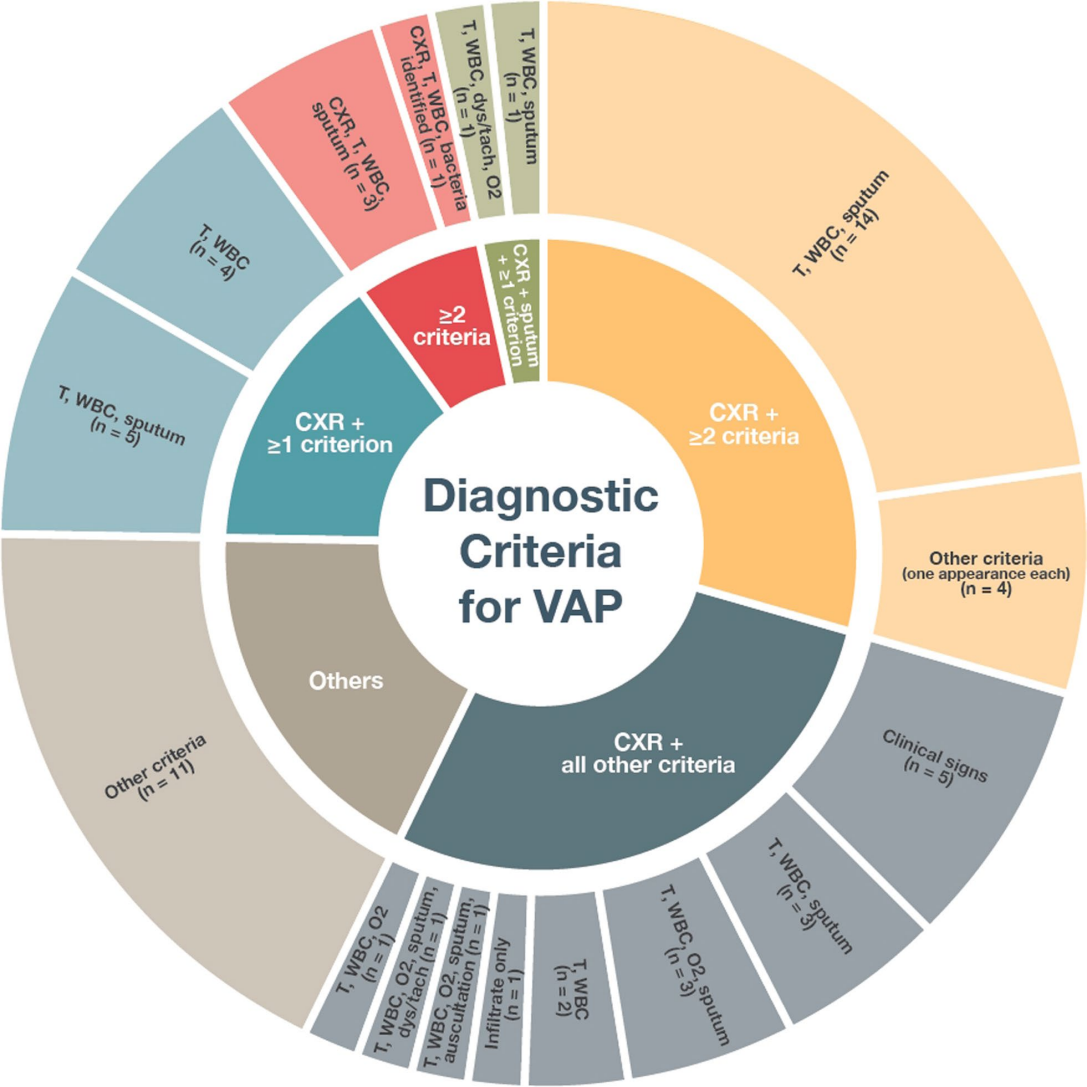
The different combinations of diagnostic criteria used in VAP RCTs. *CXR* radiological evidence of a new infiltrate; *T* temperature criterion; *WBC* white blood count criterion; *dys/tach* dyspnoea and/or tachypnoea; *O2* hypoxia; *auscultation* auscultation abnormalities

The most commonly employed set of diagnostic criteria (18/61, 29.5%) consisted of a pulmonary infiltrate along with two or more additional criteria. However, these additional criteria varied across studies (Fig. 2). A quarter (17/61) of the included studies that provided diagnostic criteria required the fulfilment of all individual criteria for diagnosis, including an infiltrate. An infiltrate and one or more additional criteria were used to establish a diagnosis of VAP in 14.8% of studies (9/61). A total of 38 different combinations of diagnostic criteria for VAP were used in the 61 identified studies. A full set of these criteria is displayed in Table S3 in the Appendix.

### Definition of “ventilator-associated”

We noted that 52.1% of included studies incorporated a specific definition of the term “ventilator-associated” (Table 4). A total of nine distinct definitions were identified across 62 RCTs. The definition most commonly used was “onset after > 48 h of mechanical ventilation” (82.3%). Other definitions employed varying time thresholds, ranging from 24 h to seven days. Additionally, certain studies introduced supplementary criteria to further delineate the concept of “ventilator-associated”, such as administration of antibiotics prior to mechanical ventilation, duration of hospitalisation, or the timing of extubation.

**Table 4:** Definition of the term “ventilator-associated” in RCTs on VAP

Definition of “ventilator-associated”	n	%
Not defined	57	47.8
Defined	62	52.1
Onset after > 48 h MV	51	82.3
Onset after > 5 d MV	3	4.8
Onset after > 3 d MV	2	3.2
Onset after > 24 h MV	1	1.6
Onset under MV	1	1.6
Onset after > 48 h MV and < 48 h prior to extubation	1	1.6
Onset after 48–72 h MV	1	1.6
Onset after > 7 d MV	1	1.6
Onset after > 96 h MV or < 96 h MV if treated with antibiotics for ≥ 5 d and hospitalised for > 7 d	1	1.6

*MV* Mechanical ventilation

## Discussion

### Summary of evidence

This systematic review provides a concise overview of the diagnostic criteria for VAP used in RCTs and the definitions attributed to the term “ventilator-associated”. A total of 119 studies on VAP, published or registered between 2010 and 2024, were included, spanning a total of 21,289 patients. The majority of studies focused exclusively on VAP, while some also included other subtypes of pneumonia alongside VAP. Diagnostic criteria were provided in only 51.2% of the studies, and the term “ventilator-associated” was defined in only 52.1% of the studies. The most commonly utilised definition for “ventilator-associated” was “onset after > 48 h of mechanical ventilation”, used by 82.3% of studies providing a definition.

In clinical practice, the diagnosis of VAP is often based on a combination of clinical signs, laboratory results, and imaging findings, yet these are not without their limitations [8]. Our systematic review revealed considerable heterogeneity among diagnostic criteria for VAP in recent RCTs. Various combinations of specific criteria were employed to define VAP, leading to significant variability. Moreover, commonly used criteria were defined in different ways, with variations observed in the thresholds set for fever/hypothermia, as well as leucocytosis/leucopoenia.

Several criteria that were used in the studies included in our review have been shown to be insufficient for confirming a diagnosis of VAP. One of the most important criteria, included in the majority of reviewed RCTs, a new or progressive pulmonary infiltrate, has previously been reported to be of limited diagnostic value due to a lack of specificity [14]. Additionally, criteria like fever/hypothermia and the measurement of biomarkers such as leukocytes, CRP, and PCT may not be effective in diagnosing or excluding VAP in various clinical settings [4, 23, 24]. Despite this, CRP is widely used and has demonstrated some clinical value in predicting VAP [25]. It is, therefore, surprising that none of the RCTs included in our review employed CRP as a diagnostic criterion.

Overall, the findings of our systematic review underline the diverse nature of VAP, with different diagnostic criteria increasing the risk of both over- and underdiagnosis of VAP [14, 26]. There have been attempts to diagnose VAP more objectively, one of these being the development of the CPIS in 1991, a six-component score that 10.9% of studies included in our review referred to [27]. This score includes different cut-offs for body temperature, leucocyte counts, tracheal secretion appearances, oxygenation levels and radiographical changes to estimate the risk for VAP. However, the CPIS has been shown not to be superior to other diagnostic criteria, and, therefore, its application remains controversial [8, 11, 22, 28]. Other commonly applied criteria, such as the surveillance-based criteria by the ECDC and CDC, did not seem to be accurate enough to detect true cases of VAP either [9–11]. Furthermore, there is limited agreement between the two surveillance-based criteria, which has previously resulted in different estimates of VAP events [29].

In lieu of definitive diagnostic scores or sets of diagnostic criteria to detect all true cases of VAP, the findings of our systematic review indicate the need for more homogeneous diagnostic criteria in future RCTs, to assure their comparability. Currently, international guidelines avoid providing clear diagnostic criteria for VAP [5, 6]. Given the significance of establishing strong consensus definitions for high-risk conditions like VAP, it is essential to emphasise even further that a uniform definition is crucial not only for advancing therapeutic research but also, and perhaps more importantly, for refining diagnostic methods. Together with core outcome sets, these definitions can help to improve the likelihood of attaining robust and reliable findings in forthcoming systematic reviews and meta-analyses [16, 30].

### Strengths and limitations

We used a comprehensive search strategy which included multiple databases and a wide range of search terms, ensuring broad identification of all potentially relevant trials. Additionally, the inclusion criteria were clearly defined, and the study selection process was conducted independently by multiple reviewers to minimise bias. The extraction sheet used for data collection was tested for inter-reviewer agreement and adapted accordingly. Another strength is the open availability of the complete dataset, maximising the transparency and reproducibility of our findings.

However, the following limitations need to be acknowledged. Firstly, the review only included RCTs conducted in English, which may have introduced language bias. This approach was adopted to ensure feasible and reliable data analysis within the scope of the resources available.

Additionally, the exclusion of studies focusing on pneumonia subtypes other than VAP may limit the generalisability of our findings. Furthermore, the lack of diagnostic criteria and definitions in a significant proportion of included studies suggests a potential reporting bias. This might be reinforced by the fact that 40.3% of data were received from trial registry platforms. Compared to final manuscript publications, reporting of eligibility criteria is often incomplete on registry platforms, therefore this must be highlighted as a limitation [31].

## Conclusions

This systematic review provides an overview of diagnostic criteria for VAP used in RCTs and the definitions attributed to the term “ventilator-associated”. Our findings highlight the heterogeneity and lack of standardisation in commonly used diagnostic criteria, as well as the variability in definitions of "ventilator-associated" across clinical trials. We emphasise the need for a uniform definition of VAP to enable better comparability between studies and interventions. The results of this review will inform the work of an upcoming task force aimed at establishing such standardised criteria.

### Supplementary Information

Additional file1 (DOCX 807 kb)

## Data Availability

Raw data are accessible via the Open Science Framework (OSF) at osf.io/v3 × 42. This link is referenced in our manuscript (Ref. 21).

## Appendix

### Search strategy

#### MEDLINE/PubMed

- #1: pneumonia [mh]
- #2: bronchopneumonia [mh]
- #3: pleuropneumonia [mh]
- #4: Healthcare-Associated Pneumonia [mh]
- #5: Ventilator-Associated Pneumonia [mh]
- #6: pneumonia [ti]
- #7: pneumonia* [ti]
- #8: bronchopneumonia [ti]
- #9: pleuropneumonia [ti]
- #10: #1 OR #2 OR #3 OR #4 OR #5 OR #6 OR #7 OR #8 OR #9
- #11: randomized controlled trial [pt]
- #12: controlled clinical trial [pt]
- #13: randomized [tiab]
- #14: placebo [tiab]
- #15: clinical trials as topic [mesh: noexp]
- #16: randomly [tiab]
- #17: trial [ti]
- #18: #11 OR #12 OR #13 OR #14 OR #15 OR #16 OR #17
- #19: animals [mh] NOT humans [mh]
- #20: children [mh] NOT adults [mh]
- #21: COVID-19 [mh] or (covid[ti]) or (coronavirus [ti]) or (sars-cov-2[ti]) or (covid-19[ti]) or (pandemic[ti])
- #22: #19 OR #20 OR #21
- #23: #18 NOT #22
- #24: #10 AND #23
- #25: Publication date: 2010 –2024

#### Cochrane library

- #1: MeSH descriptor: [Pneumonia] explode all trees
- #2: pneumonia*:ti
- #3: #1 or #2
- #4: MeSH descriptor: [COVID-19] explode all trees
- #5: COVID-19:ti
- #6: covid:ti
- #7: coronavirus:ti
- #8: sars-cov-2:ti
- #9: #4 or #5 or #6 or #7 or #8
- #10: #3 not #9
- #11: Limit: Publication Date from 2010–2024
