# Supplementary material for: Unravelling the complexity of ventilator-associated pneumonia: a systematic methodological literature review of diagnostic criteria and definitions used in clinical research
Source: Crit Care. 2024 Jul 2;28:214. doi: 10.1186/s13054-024-04991-3 (PMC11221085; doi:10.1186/s13054-024-04991-3)
Supplement: Supplementary file 1 — Additional file1 (DOCX 807 kb) [file 13054_2024_4991_MOESM1_ESM.docx]

## Additional tables and figures

| ID | Registration number | Reference |
| --- | --- | --- |
| Abdellatif 2016 | NCT02683603 | Abdellatif S, Trifi A, Daly F, Mahjoub K, Nasri R, Ben Lakhal S. Efficacy and toxicity of aerosolised colistin in ventilator-associated pneumonia: a prospective, randomised trial. Ann Intensive Care. 2016;6(1):26 |
| Abdelsalam 2018 | NA | Abdelsalam MFA, Abdalla MS, El-Abhar HSE. Prospective, comparative clinical study between high-dose colistin monotherapy and colistin-meropenem combination therapy for treatment of hospital-acquired pneumonia and ventilator-associated pneumonia caused by multidrug-resistant Klebsiella pneumoniae. J Glob Antimicrob Resist. 2018;15:127‐135. |
| Abou Warda 2022 | NCT04531332 | Abou Warda AE, Sarhan RM, Al-Fishawy HS, Moharram AN, Salem HF. Continuous Versus Intermittent Linezolid Infusion for Critically Ill Patients with Hospital-Acquired and Ventilator-Associated Pneumonia: Efficacy and Safety Challenges. Pharmaceuticals (Basel). 2022 Feb 28;15(3):296. |
| Allam 2018 | NA | Allam MGIM. The effect of use of anidulafungin on failure of weaning due to ventilator-associated pneumonia which complicate contused lungs. Open Anesthesiology Journal. 2018, 12(1), 73‐84. |
| Ammar 2018 | NA | Ammar MA, Abdalla W. Effect of extended infusion of meropenem and nebulized amikacin on Gram-negative multidrug-resistant ventilator-associated pneumonia. Saudi J Anaesth. 2018 Jan-Mar;12(1):89-94. |
| Angermair 2023 | EUCTR2012-003621-21-DE | Angermair S, Deja M, Thronicke A, Grehn C, Akbari N, Uhrig A, Asgarpur G, Spies C, Treskatsch S, Schwarz C. A prospective phase IIA multicenter double-blinded randomized placebo-controlled clinical trial evaluating the efficacy and safety of inhaled Tobramycin in patients with ventilator-associated pneumonia (iToVAP). Anaesth Crit Care Pain Med. 2023 Oct;42(5):101249. |
| Awad 2014 | NCT00210964, NCT00229008 | Awad SS, Rodriguez AH, Chuang YC, et al. A phase 3 randomized double-blind comparison of ceftobiprole medocaril versus ceftazidime plus linezolid for the treatment of hospital-acquired pneumonia. Clin Infect Dis. 2014;59(1):51‐61. |
| Aydemir 2013 | NA | Aydemir H, Akduman D, Piskin N, et al. Colistin vs. the combination of colistin and rifampicin for the treatment of carbapenem-resistant Acinetobacter baumannii ventilator-associated pneumonia. Epidemiol Infect. 2013;141(6):1214‐1222. |
| Benítez-Cano 2020 | EUCTR2016-002796-10 | Benítez-Cano A, Luque S, Sorlí L, Carazo J, Ramos I, Campillo N, Curull V, Sánchez-Font A, Vilaplana C, Horcajada JP, Adalia R, Bermejo S, Samsó E, Hope W, Grau S. Intrapulmonary concentrations of meropenem administered by continuous infusion in critically ill patients with nosocomial pneumonia: a randomized pharmacokinetic trial. Crit Care. 2020 Feb 17;24(1):55. |
| Bihan 2018 | NA | Bihan K, Zahr N, Becquemin MH, Lu X, Bertholon JF, Vezinet C, Arbelot C, Monsel A, Rouby JJ, Langeron O, Lu Q. Influence of diluent volume of colistimethate sodium on aerosol characteristics and pharmacokinetics in ventilator-associated pneumonia caused by MDR bacteria. J Antimicrob Chemother. 2018 Jun 1;73(6):1639-1646. |
| Bougle 2022 | NCT02634411 | Bouglé A, Tuffet S, Federici L, Leone M, Monsel A, Dessalle T, Amour J, Dahyot-Fizelier C, Barbier F, Luyt CE, Langeron O, Cholley B, Pottecher J, Hissem T, Lefrant JY, Veber B, Legrand M, Demoule A, Kalfon P, Constantin JM, Rousseau A, Simon T, Foucrier A; iDIAPASON Trial Investigators. Comparison of 8 versus 15 days of antibiotic therapy for Pseudomonas aeruginosa ventilator-associated pneumonia in adults: a randomized, controlled, open-label trial. Intensive Care Med. 2022 Jul;48(7):841-849. |
| Capellier 2012 | NCT01559753 | Capellier G, Mockly H, Charpentier C, et al. Early-onset ventilator-associated pneumonia in adults randomized clinical trial: comparison of 8 versus 15 days of antibiotic treatment. PLoS One. 2012;7(8):e41290. |
| ChiCTR2300069539 | ChiCTR2300069539 | NA |
| ChiCTR2300072901 | ChiCTR2300072901 | NA |
| Cisneros 2019 | NCT01292031 | Cisneros JM, Rosso-Fernández CM, Roca-Oporto C, De Pascale G, Jiménez-Jorge S, Fernández-Hinojosa E, Matthaiou DK, Ramírez P, Díaz-Miguel RO, Estella A, Antonelli M, Dimopoulos G, Garnacho-Montero J; Magic Bullet Working Group WP1. Colistin versus meropenem in the empirical treatment of ventilator-associated pneumonia (Magic Bullet study): an investigator-driven, open-label, randomized, noninferiority controlled trial. Crit Care. 2019 Nov 28;23(1):383. |
| Corrêa 2014 | RBR-86DCDX | Corrêa Rde A, Luna CM, Anjos JC, et al. Quantitative culture of endotracheal aspirate and BAL fluid samples in the management of patients with ventilator-associated pneumonia: a randomized clinical trial. J Bras Pneumol. 2014;40(6):643‐651. |
| Cousson 2015 | NCT02837835 | Cousson J, Floch T, Guillard T, et al. Lung concentrations of ceftazidime administered by continuous versus intermittent infusion in patients with ventilator-associated pneumonia. Antimicrob Agents Chemother. 2015;59(4):1905‐1909. |
| CTRI/2023/05/053239 | CTRI/2023/05/053239 | NA |
| CTRI/2023/11/059832 | CTRI/2023/11/059832 | NA |
| CTRI/2024/02/062539 | CTRI/2024/02/062539 | NA |
| De Pascale 2015 | NA | De Pascale G, Fortuna S, Tumbarello M, et al. Linezolid plasma and intrapulmonary concentrations in critically ill obese patients with ventilator-associated pneumonia: intermittent vs continuous administration. Intensive Care Med. 2015;41(1):103‐110. |
| Deans 2019 | NCT03816956 | NA |
| Drusano 2018 | NCT01570192 | Drusano GL, Corrado ML, Girardi G, Ellis-Grosse EJ, Wunderink RG, Donnelly H, Leeper KV, Brown M, Malek T, Hite RD, Ferrari M, Djureinovic D, Kollef MH, Mayfield L, Doyle A, Chastre J, Combes A, Walsh TJ, Dorizas K, Alnuaimat H, Morgan BE, Rello J, Mazo CA, Jones RN, Flamm RK, Woosley L, Ambrose PG, Bhavnani S, Rubino CM, Bulik CC, Louie A, Vicchiarelli M, Berman C. Dilution Factor of Quantitative Bacterial Cultures Obtained by Bronchoalveolar Lavage in Patients with Ventilator-Associated Bacterial Pneumonia. Antimicrob Agents Chemother. 2017 Dec 21;62(1):e01323-17. |
| Elrefaey 2020 | PACTR201909817075549 | Elrefaey BH, Zidan MS. Efficacy of manual hyperinflation on arterial blood gases in patients with ventilator-associated pneumonia. Bull Fac Phys Ther. 2020;25(1):4. |
| EUCTR2014-001406-17-NL | EUCTR2014-001406-17-NL | NA |
| EUCTR2018‐000450‐21‐ES | EUCTR2018‐000450‐21‐ES | NA |
| EUCTR2022-502229-16-00 | EUCTR2022-502229-16-00 | NA |
| Foucrier 2023 | NCT05124977 | Foucrier A, Roquilly A, Bachelet D, Martin-Loeches I, Bougle A, Timsit JF, Montravers P, Zahar JR, Eloy P, Weiss E; ASPIC study group. Antimicrobial Stewardship for Ventilator Associated Pneumonia in Intensive Care (the ASPIC trial): study protocol for a randomised controlled trial. BMJ Open. 2023 Feb 21;13(2):e065293. |
| Francois 2018 | NCT01589185 | François B, Mercier E, Gonzalez C, Asehnoune K, Nseir S, Fiancette M, Desachy A, Plantefève G, Meziani F, de Lame PA, Laterre PF, for the MASTER 1 study group. Safety and tolerability of a single administration of AR-301, a human monoclonal antibody, in ICU patients with severe pneumonia caused by Staphylococcus aureus: first-in-human trial. Intensive Care Med. 2018 Nov;44(11):1787-1796. |
| Freire 2010 | NA | Freire AT, Melnyk V, Kim MJ, et al. Comparison of tigecycline with imipenem/cilastatin for the treatment of hospital-acquired pneumonia. Diagn Microbiol Infect Dis. 2010;68(2):140‐151 |
| Frippiat 2015 | NA | Frippiat, F., Musuamba, F. T., Seidel, L., Albert, A., Denooz, R., Charlier, C., … Moutschen, M. (2014). Modelled target attainment after meropenem infusion in patients with severe nosocomial pneumonia: the PROMESSE study. Journal of Antimicrobial Chemotherapy, 70(1), 207–216. |
| Guo 2021 | NA | Guo J, Wang P, Ma X. Effect of acetylcysteine solution combined with fiberoptic bronchoscopy alveolar lavage in elderly patients with severe ventilator-associated pneumonia. Tropical Journal of Pharmaceutical Research. 2021;20(5):1061‐66. |
| Hakamifard 2021 | IRCT20171230038142N13 | Hakamifard A, Rahmani N, Homayouni A, Khorvash F, Abbasi S, Ataei B. Comparison of inhaled colistin with inhaled amikacin-fosfomycin in the treatment of ventilator-associated pneumonia caused by extensively drug-resistant (Xdr) acinetobacter: a clinical trial. Arch Clin Infect Dis. 2021, 16(1):e111084 |
| Hassan 2017 | NCT02728518 | Hassan NA, Awdallah FF, Abbassi MM, Sabry NA..Nebulized Versus IV Amikacin as Adjunctive Antibiotic for Hospital and Ventilator-Acquired Pneumonia Postcardiac Surgeries: A Randomized Controlled Trial.Crit Care Med. 2018 Jan;46(1):45-52. |
| Hellyer 2021 | NCT01972425 | Hellyer TP, McAuley DF, Walsh TS, Anderson N, Conway Morris A, Singh S, Dark P, Roy AI, Perkins GD, McMullan R, Emerson LM, Blackwood B, Wright SE, Kefala K, O'Kane CM, Baudouin SV, Paterson RL, Rostron AJ, Agus A, Bannard-Smith J, Robin NM, Welters ID, Bassford C, Yates B, Spencer C, Laha SK, Hulme J, Bonner S, Linnett V, Sonksen J, Van Den Broeck T, Boschman G, Keenan DJ, Scott J, Allen AJ, Phair G, Parker J, Bowett SA, Simpson AJ. Biomarker-guided antibiotic stewardship in suspected ventilator-associated pneumonia (VAPrapid2): a randomised controlled trial and process evaluation. Lancet Respir Med. 2020 Feb;8(2):182-191. |
| Huang 2017 | NCT00543608 | Huang DB, File TM Jr, Torres A, Shorr AF, Wilcox MH, Hadvary P, Dryden M, Corey GR. A Phase II Randomized, Double-blind, Multicenter Study to Evaluate Efficacy and Safety of Intravenous Iclaprim Versus Vancomycin for the Treatment of Nosocomial Pneumonia Suspected or Confirmed to be Due to Gram-positive Pathogens. Clin Ther. 2017 Aug;39(8):1706-1718. |
| IRCT20100107003014N26 | NA |  |
| IRCT20180802040665N2 | NA |  |
| Jaruratanasirikul 2012 | NCT01467648 | Jaruratanasirikul S, Wongpoowarak W, Kositpantawong N, Aeinlang N, Jullangkoon M. Pharmacodynamics of doripenem in critically ill patients with ventilator-associated Gram-negative bacilli pneumonia. Int J Antimicrob Agents. 2012;40(5):434‐439. |
| Jaruratanasirikul 2013 | NCT01379157 | Jaruratanasirikul S, Aeinlang N, Jullangkoon M, Wongpoowarak W. Pharmacodynamics of imipenem in critically ill patients with ventilator-associated pneumonia. J Med Assoc Thai. 2013;96(5):551‐557. |
| Jaruratanasirikul 2015 | NCT02615041 | Jaruratanasirikul S, Sriwiriyajan S, Punyo J. Comparison of the pharmacodynamics of meropenem in patients with ventilator-associated pneumonia following administration by 3-hour infusion or bolus injection. Antimicrob Agents Chemother. 2005;49(4):1337-1339. |
| JPRN-jRCT2080222278 | JPRN-jRCT2080222278 | NA |
| Khalili 2018 | IRCT201509213449N19 | Khalili H, Shojaei L, Mohammadi M, Beigmohammadi MT, Abdollahi A, Doomanlou M. Meropenem/colistin versus meropenem/ampicillin-sulbactam in the treatment of carbapenem-resistant pneumonia. J Comp Eff Res. 2018 Sep;7(9):901-911. |
| Khorvash 2019 | NA | Khorvash F, Yaghoubi S, Farsaei S, Ataei B, Hakamifard A, Mohajeri F, Gudarzi M. Comparison of two therapeutic approaches for the management of ventilator-associated pneumonia due to multidrug-resistant Acinetobacter: a randomized clinical trial study. J Immunoassay Immunochem. 2020;41(1):97-105. |
| Kollef 2012 | NCT00589693 | Kollef MH, Chastre J, Clavel M, et al. A randomized trial of 7-day doripenem versus 10-day imipenem-cilastatin for ventilator-associated pneumonia. Crit Care. 2012;16(6):R218. |
| Kollef 2017 | NCT01969799 | Kollef MH, Ricard JD, Roux D, et al. A Randomized Trial of the Amikacin Fosfomycin Inhalation System for the Adjunctive Therapy of Gram-Negative Ventilator-Associated Pneumonia: IASIS Trial. Chest. 2017;151(6):1239‐1246. |
| Kollef 2019 | NCT02070757 | Kollef MH, Nováček M, Kivistik Ü, Réa-Neto Á, Shime N, Martin-Loeches I, Timsit JF, Wunderink RG, Bruno CJ, Huntington JA, Lin G, Yu B, Butterton JR, Rhee EG. Ceftolozane-tazobactam versus meropenem for treatment of nosocomial pneumonia (ASPECT-NP): a randomised, controlled, double-blind, phase 3, non-inferiority trial. Lancet Infect Dis. 2019 Dec;19(12):1299-1311. |
| Labro 2021 | NCT02862314 | Labro G, Aptel F, Puyraveau M, Paillot J, Pili Floury S, Merdji H, Helms J, Piton G, Ecarnot F, Kuteifan K, Quenot JP, Capellier G; PROPASPI (PROcalcitonin Pneumonia/pneumonitis Associated with ASPIration) trial investigators. Impact on antimicrobial consumption of procalcitonin-guided antibiotic therapy for pneumonia/pneumonitis associated with aspiration in comatose mechanically ventilated patients: a multicenter, randomized controlled study. Ann Intensive Care. 2021 Oct 12;11(1):145. |
| Li 2021c | NA | Li Z, Xu L, Wang Y, Gao H. Comprehensive nursing intervention combined with early activityactivity applied in ventilator-associated pneumonia and its influence on blood gas index. Am J Transl Res. 2021 May 15;13(5):5647-5652. |
| Liu 2017b | NA | Liu C, Zhang YT, Peng ZY, Zhou Q, Hu B, Zhou H, Li JG. Aerosolized Amikacin as Adjunctive Therapy of Ventilator-associated Pneumonia Caused by Multidrug-resistant Gram-negative Bacteria: A Single-center Randomized Controlled Trial. Chin Med J (Engl). 2017 May 20;130(10):1196-1201. |
| Lu 2011 | NA | Lu Q, Yang J, Liu Z, et al. Nebulized ceftazidime and amikacin in ventilator-associated pneumonia caused by Pseudomonas aeruginosa. Am J Respir Crit Care Med. 2011;184(1):106‐115. |
| Makris 2018 | NA | Makris D, Petinaki E, Tsolaki V, Manoulakas E, Mantzarlis K, Apostolopoulou O, Sfyras D, Zakynthinos E. Colistin versus Colistin Combined with Ampicillin-Sulbactam for Multiresistant Acinetobacter baumannii Ventilator-associated Pneumonia Treatment: An Open-label Prospective Study. Indian J Crit Care Med. 2018 Feb;22(2):67-77. |
| Mazlan 2021 | NCT03982667 | Z Mazlan M, A H Ismail M, Ali S, Salmuna ZN, Wan Muhd Shukeri WF, Omar M. Efficacy and safety of the point-of-care procalcitonin test for determining the antibiotic treatment duration in patients with ventilator-associated pneumonia in the intensive care unit: a randomised controlled trial. Anaesthesiol Intensive Ther. 2021;53(3):207-214. |
| Meizoso 2023 | NCT05545735 | Meizoso JP, Sauaia A, Namias N, Manning RJ, Pieracci FM. Duration of Antibiotic Therapy for Early VAP Trial: Study Protocol for a Surgical Infection Society Multicenter, Pragmatic, Randomized Clinical Trial of Four versus Seven Days of Definitive Antibiotic Therapy for Early Ventilator-Associated Pneumonia in Surgical Patients. Surg Infect (Larchmt). 2023 Mar;24(2):163-168. |
| Miroliaee 2017 | IRCT2014112920134N1 | Miroliaee AE, Salamzadeh J, Shokouhi S, Sahraei Z. The study of vitamin D administration effect on CRP and Interleukin-6 as prognostic biomarkers of ventilator associated pneumonia. J Crit Care. 2018 Apr;44:300-305. |
| Mo 2024 | NCT03382548 | Mo Y, Booraphun S, Li AY, Domthong P, Kayastha G, Lau YH, Chetchotisakd P, Limmathurotsakul D, Tambyah PA, Cooper BS; REGARD-VAP investigators. Individualised, short-course antibiotic treatment versus usual long-course treatment for ventilator-associated pneumonia (REGARD-VAP): a multicentre, individually randomised, open-label, non-inferiority trial. Lancet Respir Med. 2024 May;12(5):399-408. |
| Mohamed 2018 | NA | Mohamed SS, Abdel Dayem AM, Sakr ML, Dwedar IA. The effect of administration of fosfomycin in the management of ventilator-associated pneumonia. Egypt J Chest Dis Tuberc 2018;67:318-22. |
| Momenzadeh 2022 | IRCT20150721023282N17 | Momenzadeh M, Soltani R, Shafiee F, Hakamifard A, Pourahmad M, Abbasi S. The effectiveness of colistin/levofloxacin compared to colistin/meropenem in the treatment of ventilator-associated pneumonia (VAP) caused by carbapenem-resistant Acinetobacter baumannii: a randomized controlled clinical trial. Res Pharm Sci. 2022 Dec 24;18(1):39-48. |
| Monajati 2021 | IRCT20100107003014N19 | Monajati M, Ala S, Aliyali M, Ghasemian R, Heidari F, Ahanjan M, Moradi S, Sharifpour A, Mojtahedzadeh M, Salehifar E. Clinical Effectiveness of a High Dose Versus the Standard Dose of Meropenem in Ventilator-associated Pneumonia Caused by Multidrugresistant Bacteria: A Randomized, Single-blind Clinical Trial. Infect Disord Drug Targets. 2021;21(2):274-283. |
| Mosaed 2018 | IRCT20120703010178N15 | Mosaed R, Haghighi M, Kouchak M, Miri MM, Salarian S, Shojaei S, Javadi A, Taheri S, Nazirzadeh P, Foroumand M, Sistanizad M. Interim Study: Comparison Of Safety And Efficacy of Levofloxacin Plus Colistin Regimen With Levofloxacin Plus High Dose Ampicillin/Sulbactam Infusion In Treatment of Ventilator-Associated Pneumonia Due To Multi Drug Resistant Acinetobacter. Iran J Pharm Res. 2018;17(Suppl2):206-213. |
| Nassar 2018 | NA | Nassar YS, Saber-Ayad M, Shash RY. Combined microbiological and clinical outcomes of short-term inhaled colistin adjunctive therapy in ventilator-associated pneumonia. Egypt J Chest Dis Tuberc 2018;67:376-83. |
| NCT00396578 | NCT00396578 | NA |
| NCT01356472 | NCT01356472 | NA |
| NCT01853982 | NCT01853982 | NA |
| NCT01865266 | NCT01865266 | NA |
| NCT01994980 | NCT01994980 | NA |
| NCT02459158 | NCT02459158 | NA |
| NCT02478710 | NCT02478710 | NA |
| NCT02574130 | NCT02574130 | NA |
| NCT02897466 | NCT02897466 | NA |
| NCT02906722 | NCT02906722 | NA |
| NCT03006679 | NCT03006679 | NA |
| NCT03027609 | NCT03027609 | NA |
| NCT03121690 | NCT03121690 | NA |
| NCT03409679 | NCT03409679 | NA |
| NCT03477292 | NCT03477292 | NA |
| NCT03581370 | NCT03581370 | NA |
| NCT03582007 | NCT03582007 | NA |
| NCT03583333 | NCT03583333 | NA |
| NCT03622450 | NCT03622450 | NA |
| NCT03711331 | NCT03711331 | NA |
| NCT03891212 | NCT03891212 | NA |
| NCT03921645 | NCT03921645 | NA |
| NCT04438187 | NCT04438187 | NA |
| NCT04467892 | NCT04467892 | NA |
| NCT04633317 | NCT04633317 | NA |
| NCT05204563 | NCT05204563 | NA |
| NCT05230472 | NCT05230472 | NA |
| NCT05354778 | NCT05354778 | NA |
| NCT05685615 | NCT05685615 | NA |
| NCT05696093 | NCT05696093 | NA |
| NCT05843786 | NCT05843786 | NA |
| NCT05952648 | NCT05952648 | NA |
| NCT05989269 | NCT05989269 | NA |
| NCT06087536 | NCT06087536 | NA |
| NCT06238297 | NCT06238297 | NA |
| NCT06370598 | NCT06370598 | NA |
| Niederman 2012 | NCT01004445 | Niederman MS, Chastre J, Corkery K, Fink JB, Luyt CE, García MS. BAY41-6551 achieves bactericidal tracheal aspirate amikacin concentrations in mechanically ventilated patients with Gram-negative pneumonia. Intensive Care Med. 2012;38(2):263‐271. |
| Niederman 2020 | NCT01799993; NCT00805168 | Niederman MS, Alder J, Bassetti M, Boateng F, Cao B, Corkery K, Dhand R, Kaye KS, Lawatscheck R, McLeroth P, Nicolau DP, Wang C, Wood GC, Wunderink RG, Chastre J. Inhaled amikacin adjunctive to intravenous standard-of-care antibiotics in mechanically ventilated patients with Gram-negative pneumonia (INHALE): a double-blind, randomised, placebo-controlled, phase 3, superiority trial. Lancet Infect Dis. 2020 Mar;20(3):330-340. |
| Papazian 2013 | NCT01057758 | Papazian L, Roch A, Charles PE, Penot-Ragon C, Perrin G, Roulier P, Goutorbe P, Lefrant JY, Wiramus S, Jung B, Perbet S, Hernu R, Nau A, Baldesi O, Allardet-Servent J, Baumstarck K, Jouve E, Moussa M, Hraiech S, Guervilly C, Forel JM; STATIN-VAP Study Group. Effect of statin therapy on mortality in patients with ventilator-associated pneumonia: a randomized clinical trial. JAMA. 2013 Oct 23;310(16):1692-700. |
| Poole 2022 | 10.5258/SOTON/P0070 | Poole S, Tanner AR, Naidu VV, Borca F, Phan H, Saeed K, Grocott MPW, Dushianthan A, Moyses H, Clark TW. Molecular point-of-care testing for lower respiratory tract pathogens improves safe antibiotic de-escalation in patients with pneumonia in the ICU: Results of a randomised controlled trial. J Infect. 2022 Dec;85(6):625-633. |
| Pourheidar 2019 | IRCT20120703010178N18 | Pourheidar E, Haghighi M, Kouchek M, Miri MM, Shojaei S, Salarian S, Hassanpour R, Sistanizad M. Comparison of Intravenous Ampicillin-sulbactam Plus Nebulized Colistin with Intravenous Colistin Plus Nebulized Colistin in Treatment of Ventilator Associated Pneumonia Caused by Multi Drug Resistant Acinetobacter Baumannii: Randomized Open Label Trial. Iran J Pharm Res. 2019 Fall;18(Suppl1):269-281. |
| Qin 2018 | NA | Qin Y, Zhang J, Wu L, Zhang D, Fu L, Xue X.Comparison of the treatment efficacy between tigecycline plus high-dose cefoperazone-sulbactam and tigecycline monotherapy against ventilator-associated pneumonia caused by extensively drug-resistant Acinetobacter baumannii. Int J Clin Pharmacol Ther. 2018 Mar;56(3):120-129. |
| Ramirez 2013 | NCT00707239 | Ramirez J, Dartois N, Gandjini H, Yan JL, Korth-Bradley J, McGovern PC. Randomized phase 2 trial to evaluate the clinical efficacy of two high-dosage tigecycline regimens versus imipenem-cilastatin for treatment of hospital-acquired pneumonia. Antimicrob Agents Chemother. 2013;57(4):1756‐1762. |
| Rattanaumpawan 2010 | NCT00920270 | Rattanaumpawan P, Lorsutthitham J, Ungprasert P, Angkasekwinai N, Thamlikitkul V.Randomized controlled trial of nebulized colistimethate sodium as adjunctive therapy of ventilator-associated pneumonia caused by Gram-negative bacteria. J Antimicrob Chemother. 2010 Dec;65(12):2645-9. |
| Razzazzadeh 2022 | IRCT20130917014693N12 | Razzazzadeh S, Darazam IA, Hajiesmaeili M, Salamzadeh J, Mahboubi A, Sadeghnezhad E, Sahraei Z. Investigation of pharmacokinetic and clinical outcomes of various meropenem regimens in patients with ventilator-associated pneumonia and augmented renal clearance. Eur J Clin Pharmacol. 2022 May;78(5):823-829. |
| Rubinstein 2011 | NCT00107952, NCT00124020 | Rubinstein E, Lalani T, Corey GR, Kanafani ZA, Nannini EC, Rocha MG, Rahav G, Niederman MS, Kollef MH, Shorr AF, Lee PC, Lentnek AL, Luna CM, Fagon JY, Torres A, Kitt MM, Genter FC, Barriere SL, Friedland HD, Stryjewski ME; ATTAIN Study Group. Telavancin versus vancomycin for hospital-acquired pneumonia due to gram-positive pathogens. Clin Infect Dis. 2011 Jan 1;52(1):31-40. |
| Schmelzer 2013 | NA | Schmelzer TM, Christmas AB, Norton HJ, Heniford BT, Sing RF. Vancomycin intermittent dosing versus continuous infusion for treatment of ventilator-associated pneumonia in trauma patients. Am Surg. 2013;79(11):1185‐1190. |
| Shahrokhi 2023 | IRCT20181105041567N1 | Shahrokhi M, Gholizadeh Gerdrodbari M, Mousavi SM, Rastaghi S, Enayati F. Comparing the Effect of Respiratory Physiotherapy and Positive End-Expiratory Pressure Changes on Capnography Results in Intensive Care Unit Patients with Ventilator-Associated Pneumonia. Tanaffos. 2023 Mar;22(3):298-304. |
| Stokker 2015 | NCT02440828 | Stokker J, Karami M, Hoek R, Gommers D, van der Eerden M. Effect of adjunctive tobramycin inhalation versus placebo on early clinical response in the treatment of ventilator-associated pneumonia: the VAPORISE randomized-controlled trial. Intensive Care Med. 2020 Mar;46(3):546-548. |
| Titov 2021 | NCT02493764 | Titov I, Wunderink RG, Roquilly A, Rodríguez Gonzalez D, David-Wang A, Boucher HW, Kaye KS, Losada MC, Du J, Tipping R, Rizk ML, Patel M, Brown ML, Young K, Kartsonis NA, Butterton JR, Paschke A, Chen LF. A Randomized, Double-blind, Multicenter Trial Comparing Efficacy and Safety of Imipenem/Cilastatin/Relebactam Versus Piperacillin/Tazobactam in Adults With Hospital-acquired or Ventilator-associated Bacterial Pneumonia (RESTORE-IMI 2 Study). Clin Infect Dis. 2021 Dec 6;73(11):e4539-e4548. |
| Torres 2019 | NCT01808092 | Torres A, Rank D, Melnick D, Rekeda L, Chen X, Riccobene T, Critchley IA, Lakkis HD, Taylor D, Talley AK. Randomized Trial of Ceftazidime-Avibactam vs Meropenem for Treatment of Hospital-Acquired and Ventilator-Associated Bacterial Pneumonia (REPROVE): Analyses per US FDA-Specified End Points. Open Forum Infect Dis. 2019 Apr 25;6(4):ofz149. |
| UMIN000003972 | UMIN000003972 | NA |
| Wu 2021 | NA | Wu L, Liu B. The clinical effect of a bronchofiberscope in treating severe ventilator-associated pneumonia. Am J Transl Res. 2021 Jun 15;13(6):6966-6972. |
| Wunderink 2012 | NA | Wunderink RG, Niederman MS, Kollef MH, Shorr AF, Kunkel MJ, Baruch A, McGee WT, Reisman A, Chastre J. Linezolid in methicillin-resistant Staphylococcus aureus nosocomial pneumonia: a randomized, controlled study. Clin Infect Dis. 2012 Mar 1;54(5):621-9. |
| Wunderink 2021 | NCT02019420 | Wunderink RG, Roquilly A, Croce M, Rodriguez Gonzalez D, Fujimi S, Butterton JR, Broyde N, Popejoy MW, Kim JY, De Anda C. A Phase 3, Randomized, Double-Blind Study Comparing Tedizolid Phosphate and Linezolid for Treatment of Ventilated Gram-Positive Hospital-Acquired or Ventilator-Associated Bacterial Pneumonia. Clin Infect Dis. 2021 Aug 2;73(3):e710-e718. |
| Xu 2021 | ChiCTR2100048112 | Xu Z, Chen J, Xu R. A randomised controlled study: efficacy of ICU nursing risk management combined with the cluster nursing model and its effect on quality of life and inflammatory factor levels of patients with acute respiratory distress syndrome and ventilator-associated pneumonia. Ann Palliat Med. 2021 Jul;10(7):7587-7595. |
| Yoshimura 2018 | NCT03506113 | Yoshimura J, Yamakawa K, Kinoshita T, Ohta Y, Morimoto T. GRam stain-guided Antibiotics ChoicE for Ventilator-Associated Pneumonia (GRACE-VAP) trial: rationale and study protocol for a randomised controlled trial. Trials. 2018 Nov 8;19(1):614. |
| Zhang 2021 | NA | Zhang HW, Wei LY, Wang JX, Liu SZ, Xing D, Zhang R, Guo H, Chen LJ, Zhang J. Effect of traditional Chinese preparation Xuebijing on inflammatory markers in patients with ventilator-associated pneumonia. Journal of Acute Disease 10(5):p 216-220, October 2021. |

**Table S1. The included studies on VAP**. NA – not available.


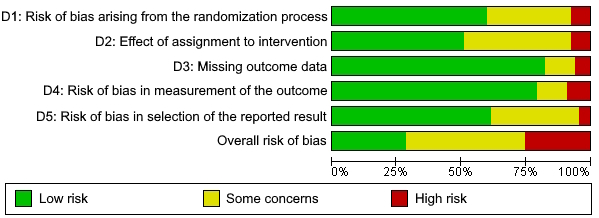


**Figure SF1. The overall risk of bias in 67 RCTs on VAP with published results.** The tool used to assess risk of bias was RoB-2 (D1-5 – RoB-2 domains 1-5).

**
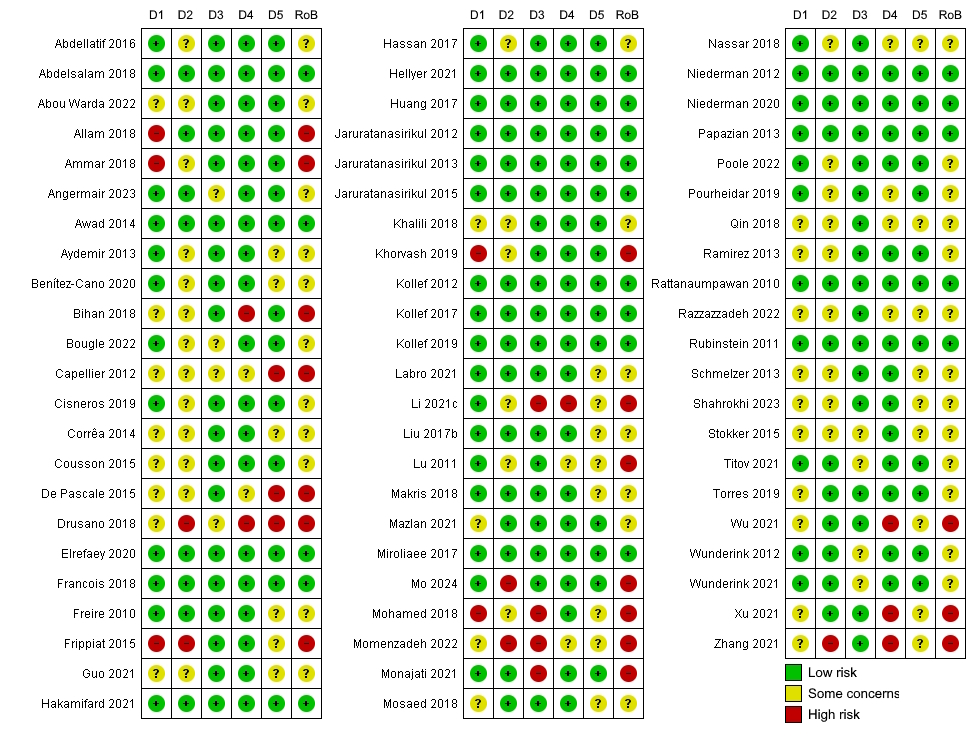
**

**Figure SF2. The details regarding risk of bias in 67 RCTs on VAP with published results.** The tool used to assess risk of bias was RoB-2 (D1-5 – RoB-2 domains 1-5, RoB: overall risk of bias).

| **Site of temperature measurement mentioned** | **n** |
| --- | --- |
| Total | 16 |
| Axillary | 4 |
| Oral | 6 |
| Rectal | 2 |
| Core | 3 |
| Forehead | 1 |

**Table S2. Site of temperature measurement**.

| **Diagnostic criteria in studies with a definition** | | **n** | **%** |
| --- | --- | --- | --- |
| All criteria | Criteria: fever/hypothermia, leucocytosis/leucopoenia, bacteria identified, respiratory failure | 1 | 1.6 |
| Infiltrate and all other criteria | Total | 17 | 27.9 |
|  | Criteria: clinical signs | 5 |  |
|  | Criteria: fever, leucocytosis, hypoxia, sputum | 2 |  |
|  | Criteria: fever/hypothermia, leucocytosis/leucopoenia, dyspnoea, hypoxia, cough, sputum | 1 |  |
|  | Criteria: fever/hypothermia, leucocytosis/leucopoenia, hypoxia, sputum, auscultation abnormalities | 1 |  |
|  | Criteria: fever/hypothermia, leucocytosis/leucopoenia | 1 |  |
|  | Criteria: fever, leucocytosis/leucopoenia | 1 |  |
|  | Criteria: fever/hypothermia, leucocytosis, sputum | 1 |  |
|  | Criteria: fever, leucocytosis/leucopoenia, hypoxia | 1 |  |
|  | Criteria: fever, leucocytosis/leucopoenia, hypoxia, sputum | 1 |  |
|  | Criteria: fever/hypothermia, leucocytosis/leucopoenia, sputum | 1 |  |
|  | Criteria: only infiltrate mentioned | 1 |  |
|  | Criteria: fever, leucocytosis, sputum | 1 |  |
| ≥2 criteria | Total | 4 | 6.6 |
|  | Criteria: infiltrate, fever/hypothermia, leucocytosis/leucopoenia, sputum | 2 |  |
|  | Criteria: infiltrate, fever, leucocytosis/leucopoenia, sputum | 1 |  |
|  | Criteria: infiltrate, fever/hypothermia, leucocytosis/leucopoenia, bacteria identified | 1 |  |
| Infiltrate and ≥1 criterion | Total | 9 | 14.8 |
|  | Criteria: fever, leucocytosis, sputum | 4 |  |
|  | Criteria: fever/hypothermia, leucocytosis/leucopoenia | 4 |  |
|  | Criteria: fever/hypothermia, leucocytosis/leucopoenia, sputum | 1 |  |
| Infiltrate and ≥2 criteria | Total | 18 | 29.5 |
|  | Criteria: fever, leucocytosis/leucopoenia, sputum | 7 |  |
|  | Criteria: fever/hypothermia, leucocytosis/leucopoenia, sputum | 6 |  |
|  | Criteria: fever, leucocytosis, sputum | 1 |  |
|  | Criteria: fever, leucocytosis, sputum, bacteria identified | 1 |  |
|  | Criteria: fever/hypothermia, leucocytosis/leucopoenia, hypoxia, sputum | 1 |  |
|  | Criteria: fever/hypothermia, leucocytosis/leucopoenia, dyspnoea, tachypnoea, hypoxia, cough, sputum, auscultation abnormalities | 1 |  |
|  | Criteria: dyspnoea, tachypnoea, hypoxia, cough, sputum, auscultation abnormalities | 1 |  |
| Infiltrate and sputum and ≥1 other criterion | Total | 2 | 3.8 |
|  | Criteria: fever/hypothermia, leucocytosis/leukopenia, sputum | 1 |  |
|  | Criteria: fever/hypothermia, leucocytosis/leukopenia, dyspnoea, tachypnoea, hypoxia | 1 |  |
| Infiltrate and hypoxia and ≥1 other criterion | Criteria: fever/hypothermia, leucocytosis/leukopenia | 1 | 1.6 |
| Infiltrate and hypoxia and bacteria identified and ≥1 other criterion | Criteria: fever/hypothermia, leucocytosis/leukopenia | 1 | 1.6 |
| Infiltrate and sputum and ≥1 criterion of set 1 and ≥1 criterion of set 2 | Total | 1 | 1.6 |
|  | Criteria set 1: fever, leucocytosis/leucopoenia, mental status |  | |
|  | Criteria set 2: sputum, dyspnoea/tachypnoea, auscultation abnormalities, hypoxia |  |  |
| Infiltrate and sputum and ≥1 criterion of set 1 and ≥2 criteria of set 2 | Total | 1 | 1.6 |
|  | Criteria set 1: fever, leucocytosis/leucopoenia |  | |
|  | Criteria set 2: sputum, tachypnoea, hypoxia |  |  |
| Infiltrate and ≥1 criterion of set 1 and ≥1 criterion of set 2 | Total | 1 | 1.6 |
|  | Criteria set 1: respiratory symptoms, hypoxia, sputum |  | |
|  | Criteria set 2: fever/hypothermia, leucocytosis/leukopenia |  |  |
| Infiltrate and ≥1 criterion of set 1 and ≥2 criteria of set 2 | Total | 1 | 1.6 |
|  | Criteria set 1: fever/hypothermia, leucocytosis/leukopenia |  | |
|  | Criteria set 2: respiratory failure, sputum, tachypnoea, dyspnoea, auscultation abnormalities, hypoxia |  |  |
| Infiltrate and ≥2 criteria of set 1 and ≥2 criteria of set 2 | Total | 1 | 1.6 |
|  | Criteria set 1: cough, sputum, auscultation abnormalities, dyspnoea, tachypnoea, hypoxia |  | |
|  | Criteria set 2: fever/hypothermia, tachypnoea, tachycardia, mental status, leucocytosis/leucopoenia |  |  |
| Infiltrate and (bacteria identified or ≥2 criterion of set 1) and ≥2 criteria of set 2 | Total | 1 | 1.6 |
|  | Criteria set 1: cough, sputum, auscultation abnormalities, dyspnoea/tachypnoea, hypoxemia |  | |
|  | Criteria set 2: fever/hypothermia, tachypnoea, tachycardia, mental status, leucocytosis/leucopoenia |  |  |
| Infiltrate and (fever or leucocytosis/leucopoenia) and ≥2 other criteria | Criteria: dyspnoea, tachypnoea, cough, sputum, chest pain, auscultation abnormalities | 1 | 1.6 |
| ≥1 criterion of set 1 and ≥1 criterion of set 2 | Total | 1 | 1.6 |
|  | Criteria set 1: hypoxia, sputum, ventilator changes |  | |
|  | Criteria set 2: fever/hypothermia, leucocytosis/leukopenia |  |  |

**Table S3. Sets of criteria used to diagnose VAP.**
